# A Shift in the Biogenic Silica of Sediment in the Larsen B Continental Shelf, Off the Eastern Antarctic Peninsula, Resulting from Climate Change

**DOI:** 10.1371/journal.pone.0052632

**Published:** 2013-01-02

**Authors:** Elisabet Sañé, Enrique Isla, María Ángeles Bárcena, David J. DeMaster

**Affiliations:** 1 Department of Marine Geology, Institute of Marine Sciences (ICM-CSIC), Barcelona, Spain; 2 Department of Geology and Paleontology, University of Salamanca, Salamanca, Spain; 3 Department of Marine, Earth and Atmospheric Sciences, North Carolina State University, Raleigh, North Carolina, United States of America; Plymouth University, United Kingdom

## Abstract

In 2002, section B of the Larsen ice shelf, off of the Eastern Antarctic Peninsula, collapsed and created the opportunity to study whether the changes at the sea surface left evidence in the sedimentary record. Biogenic silica is major constituent of Antarctic marine sediment, and its presence in the sediment column is associated with diatom production in the euphotic zone. The abundance of diatom valves and the number of sponge spicules in the biogenic silica was analyzed to determine how the origin of the biogenic silica in the upper layers of the sediment column responded to recent environmental changes. Diatom valves were present only in the upper 2 cm of sediment, which roughly corresponds to the period after the collapse of the ice shelf. In contrast, sponge spicules, a more robust form of biogenic silica, were also found below the upper 2 cm layer of the sediment column. Our results indicate that in this region most of the biogenic silica in the sedimentary record originated from sponge spicules rather than diatoms during the time when the sea surface was covered by the Larsen ice shelf. Since the collapse of the ice shelf, the development of phytoplankton blooms and the consequent influx of diatom debris to the seabed have shifted the biogenic silica record to one dominated by diatom debris, as occurs in most of the Antarctic marine sediment. This shift provides further evidence of the anthropogenic changes to the benthic habitats of the Antarctic and will improve the interpretation of the sedimentary record in Polar Regions where these events occur.

## Introduction

Over the last 60 years, atmospheric and oceanic temperatures in the Antarctic Peninsula have increased more than the global average [1], [2]. This increase is responsible for the retreat of the ice shelf on both sides of the peninsula [3] and for the collapse of the Larsen B ice shelf, which disintegrated in 2002 after millennia of stability [4]. The collapse of the Larsen B ice shelf on the east side of the Antarctic Peninsula must have changed the conditions in the water column, which now favor increased primary production in the euphotic zone [5]. The sediment cores collected on the continental shelf below the collapsed sections of the ice shelf contained phytopigments and diatom valves together with excess ^210^Pb activity, only in the upper 2 cm of the sediment column, which were attributed to a recent flux of biogenic material to the seafloor [6], [7]. ^210^Pb reaches the marine environment by atmospheric precipitation and in-situ decay of its parent, ^226^Ra. ^210^Pb is insoluble in sea water and is scavenged from the water column by settling particles during their transit to the seabed. Upon settlement, these particles produce “excess” ^210^Pb activity which adds to the supported activity levels found deeper in the sediment column, where the older material has been stabilized.

Along with diatoms, radiolaria, siliceous sponges and silicoflagellates contribute their siliceous hard parts to the biogenic silica that reaches the seafloor [8], [9], [10]. In general, diatom valves, radiolaria and sponge spicules are the principal sources of biogenic silica in marine sediments [11], [9], [12], [13]. Southern Ocean sediments account for ∼50% of the biogenic silica deposited in the marine environment, and most of this siliceous material has been attributed to diatoms [10]. Diatoms can have high biomass in Antarctic coastal regions [14], [15] and can represent as much as 40% of the total primary productivity in the Southern Ocean [16]. Despite partial degradation during grazing [17] and dissolution in the water column and seabed [18], siliceous diatom frustules are often well-preserved in the sediment column and can serve as an indicator of the environmental conditions at the time that they were formed [19], [20]. Siliceous sponges are often important components of Antarctic benthic communities [21]. Most Demospongiae and Hexactinellida sponges produce siliceous skeletons composed of individualized elements (spicules) with lengths ranging from micrometers to centimeters [22]. Hexactinellids are one of the dominant groups in the benthic communities of the Weddell Sea [23] and play an important role in forming substrata by generating spicule mats [24], [23]. Some spicules are too fragile to survive intact in the sedimentary record, which prevents them from serving as accurate oceanographic proxies of the paleoenvironmental record. However, sponges can be important contributors to biogenic silica in the sedimentary column because they are relatively long-lived and their spicules dissolve more slowly than diatom frustules [13], [25]. Earlier observations in the Larsen region suggested that hexactinellid sponges dwelled on the continental shelf before the collapse of the Larsen ice shelf [26]. Thus, it is possible that sponge spicules could have constituted an important fraction of the biogenic silica in the sediment column of that region during the period when diatom development was restricted by the ice shelf [7]. The recent disintegration of the Larsen ice shelf offers a unique opportunity for research. The collapse of the ice shelf and the development of diatom blooms may have changed the sedimentary regime of biogenic silica from one dominated by sponge spicules to one in which diatom frustules play a greater role.

## Methods

### 1. Sediment sampling

**Figure 1:**
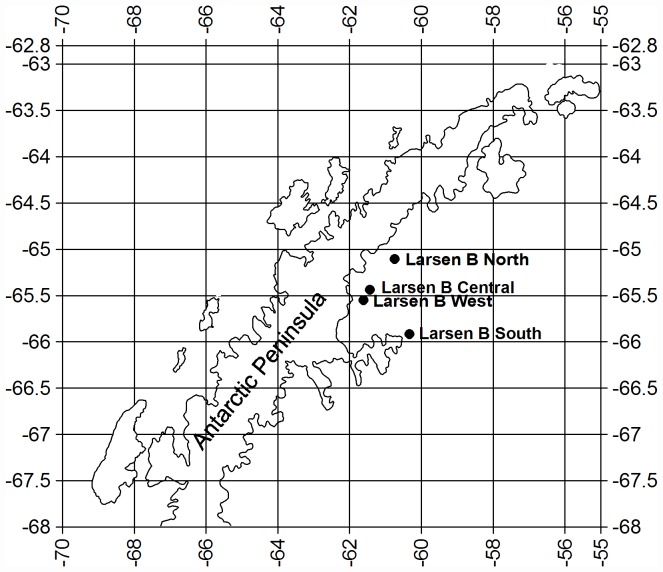
Study area with the four sampling stations.

In the austral summer of 2006–2007, during ANT-XXIII/8, the R/V *Polarstern* reached the area off the Eastern Antarctic Peninsula (EAP) that was previously occupied by section B of the Larsen ice shelf. Four sediment cores were collected using a multi- corer with a diameter of 10 cm [27] at the Larsen B South (LBS), Larsen B West (LBW), Larsen B Central (LBC) and Larsen B North (LBN) stations (**Fig. 1**). Before laboratory and microscopic analyses, the samples were freeze dried (at 0.1 mbar and −80°C) for 24 hours. Permission to collect samples from the prospected areas was obtained from the Spanish Polar Committee (Ref. CPE-EIA-2006-16) observing the guidelines of the Antarctic Treaty. None of the sampling stations occupied specially protected areas in the Antarctic.

### 2. Biogenic silica

The percentage of biogenic silica (SiO_2_) in the sediment (expressed as dry weight %) was measured following established procedures [28], [9]. Five milliliters of a 10% hydrogen peroxide (H_2_O_2_) solution was added to approximately 100–200 mg of dry sediment to remove the organic matter. After 30 minutes (min), 5 ml of a 10% hydrochloric acid (HCl) solution was added to dissolve the calcium carbonate (CaCO_3_). The samples were rinsed with bi-distilled water and, after centrifugation (5 min at 5000 revolutions per minute), were dried in the oven before alkaline extraction. After sonication for 5 min in 40 ml of a 2 M sodium carbonate (Na_2_CO_3_) solution, the samples were placed in a bath (T = 85°C) for 5 h. Five milliliters of solution were collected twice, after 2 h and again after 5 h of extraction. The extraction after 2 h primarily targeted biogenic SiO_2_, whereas the extraction after 5 h primarily targeted the lithogenic silica minerals that took longer to dissolve in Na_2_CO_3_ [9]. After extraction, 17.5 ml of a ammonium molybdate tetrahydrate (NH_4_)6Mo_7_O_24_ 4H_2_O) solution was added to every aliquot, and after 20 min, 7.5 ml of a reducing solution with sodium metol-sulfite (C_7_H_10_NO)_2_SO_4_ Na_2_SO_3_), oxalic acid (C_2_H_2_O_4_) and sulfuric acid (H_2_SO_4_) were added to produce a blue chromophore, which was read using a spectrophotometer at 815 nm. A regression line was plotted through the two SiO_2_ concentration values corresponding to the 2 h and 5 h extractions. The intercept of the regression line with the y-axis corresponded to the percent weight of biogenic SiO_2_ in the sample [9].

### 3. Diatom valves and sponge spicules

Diatom valves and sponge spicules were counted using an optical microscope. HCl and H_2_O_2_ were added to the dry sediment to dissolve carbonates and attack organic matter, respectively. The sediment was rinsed several times with bi-distilled water, the slides were mounted, and diatom valve counts were performed at 1000 magnification using a Leica DMLB with phase-contrast illumination. The counts were carried out on permanent slides of acid-cleaned material (Permount mounting medium). The recommendations of [29] were followed for counting microfossil valves. Several transects along each cover slip were examined according to the abundance of the diatoms. A minimum of 350 valves, including at least 100 valves from non-dominant taxa, were counted per sample. In addition to diatoms, sponge spicules were also counted.

To calculate the contribution of diatoms and siliceous sponges to the biogenic silica, we estimated the weight of the main groups of diatom taxa and siliceous spicules. We calculated the volume of specimen types of *Chaetoceros* resting spores (RS) (approximately 43% of the diatom assemblage), *Fragilariopsis curta* (the primary species of the Sea-ice taxa group, representing approximately 45% of the assemblage) and of *Thalassosira antarctica* RS (a minor component of the assemblage). For this task, we used NIS- Element BR3.1 software for microscope image analyses with a NIKON Eclipse 80 at x1000 magnification. The mean volume of a diatom valve and a sponge spicule fragment were calculated by measuring 100 specimens for each sample. The volumes were multiplied by the biogenic silica density, which was assumed to be ∼2 g cm^−3^ [30]. For *Chaetoceros* RS, the mean value of the volume occupied by biogenic SiO_2_ was 3.7×10^−11^ cm^3^ diatom^−1^, and the weight of the biogenic SiO_2_ was 7.4×10^−11^ grams per diatom. Assuming that approximately 30% of the surface of a *F. curta* specimen-type was empty due to the perforations in the structure of the valve, the mean volume occupied by biogenic silica was 4.41×10^−11^ cm^3^ diatom^−1^. The estimated weight of the *F. curta* specimen-type was 8.82×10^−11^ grams of biogenic SiO_2_ per diatom. In the case of *T. antarctica* RS, assuming that approximately 50% of the valve surface was perforated, the silica-volume for the specimen-type was 9.82×10^−11^ cm^3^ diatom^−1^. Consequently, the estimated weight of the specimen type was 1.96×10^−11^ grams of biogenic SiO_2_ per diatom.

The morphometrics for sponge spicules consisted of the length, external diameter and internal diameter of the fragments. The difference between the external and internal cylinders was considered to be the silica-volume of the fragment type, which was 2.39×10^−9^ cm^3^ spicule^−1^. Using the same value of 2 g cm^−3^ for biogenic silica, we determined that the weight of the biogenic SiO_2_ of a sponge spicule fragment was 4.78×10^−9^ grams. To obtain the total amount of biogenic silica originating from diatoms and sponge spicules, the weight in grams of the biogenic SiO_2_ per diatom and sponge spicule fragments was multiplied by the abundance of diatoms and spicule fragments, respectively.

## Results

### 1. Biogenic silica

**Figure 2:**
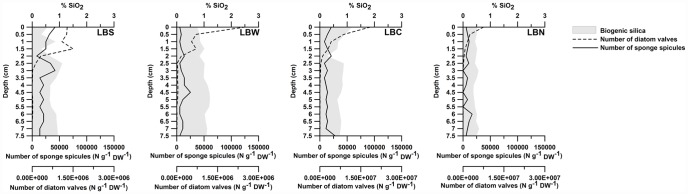
In the superior x-axis, percentage of biogenic silica from silica extractions. In the inferior x-axis, number of diatom valves and number of sponge spicules. Axis scale for diatom valves is different for Larsen B Central (LBC) and North (LBN).

In the surface sediment (upper 0.5 cm), the biogenic SiO_2_ content varied between 0.53% (LBN) and 1.2% (LBC) (**Fig. 2**). The percentage of biogenic silica did not decrease with depth in any of the four cores (**Fig. 2**).

### 2. Diatom valves and sponge spicules

The number of diatom valves in the surface sediment varied between ∼1.3×10^6^ valves per gram of dry sediment (N g DW^−1^) (station LBS) and ∼18×10^6^ valves g DW^−1^ (station LBC) (**Fig. 2**). In all of the cores, the diatom valve abundances decreased with depth and were negligible at depths greater than 2 cm (**Fig. 2**).

The number of sponge spicules in the upper 0.5 cm varied between ∼5600 (LBW and LBN) and ∼42000 (LBS) spicules g DW^−1^ (**Fig. 2**). The number of sponge spicules did not decrease with depth in any of the cores (**Fig. 2**).

**Figure 3:**
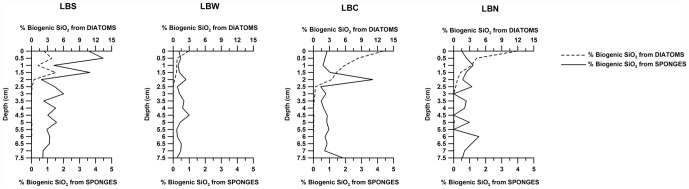
In the superior x-axis, percentage of biogenic silica from diatoms (calculated from the number of frustules and associated weight) and, in the inferior x-axis, percentage of biogenic silica from sponges (calculated from the number of spicules and associated weight), as a function of depth.

In the surface sediment (0–0.5 cm depth), the biogenic silica content from diatoms varied between 0.1×10^−3^ (LBS) and 1.5×10^−3^ (LBC) grams of biogenic silica per gram of dry sediment (**Fig. 3**). Below 2 cm, the concentration of biogenic silica from diatoms was negligible (**Fig. 3**). The contribution of sponge spicules to the biogenic silica varied between 0.03×10^−3^ (LBW) and 0.2×10^−3^ (LBS) grams of biogenic silica per gram of dry sediment (**Fig. 3**)

## Discussion

### 1. Biogenic silica under ice shelves

Overall, the biogenic silica percentages obtained in the present study were rather low (**Fig. 2**) for the Antarctic context (including the Weddell and Ross Seas and the Bransfield and Gerlache Straits), where dry weight percentages varied between 1% and 49% in surface sediments [31], [32], [33], [34]. Based on the frustule and spicule volumes and the biogenic silica density, less than ∼14% (LBC station, from 0 to 0.5 cm depth) of the total biogenic silica in our samples was from diatoms or sponge spicules (**Fig. 3**). These low values suggest that significant amounts of silica leached from coexisting aluminosilicates in the lithogenic fraction of the sample, which could not be identified with the alkaline extraction method. However, alkaline extraction is the standard technique used to measure biogenic silica in marine sediments and therefore allows a direct comparison of the biogenic silica contents of our samples to those of different Antarctic regions [31], [32], [33], [34]. The methodological restrictions led us to use the diatom frustule and spicule concentrations (**Fig. 2** and **Fig. 3**) to establish reliable comparisons between the sites and to identify changes in the sedimentary regime of biogenic silica within the study area. Even given the potential overestimation from the aluminum silicates, there was relatively little biogenic silica in the continental shelf sediments below the extinct Larsen ice shelf. The concentrations indicate that the supply of biogenic silica to the seabed below the ice shelves is comparatively low and that this condition persists a decade after the collapse of the Larsen ice shelf. This persistence most likely occurs because the sea surface in the Larsen region still has heavy sea ice during most of the year, which prevents primary productivity from reaching the levels that are reached truly open water. However, the exponential increase in diatomaceous material (**Fig. 2**) towards the surface of the sediment cores indicates that the production of diatomaceous biogenic silica is increasing. It is worth mentioning that station LBC is found in the axis of a glacial trough, where sediment focusing produces higher concentrations of diatom valves and biogenic silica percentages (**Fig. 2**).

### 2. Spicules as source of biogenic silica

**Figure 4:**
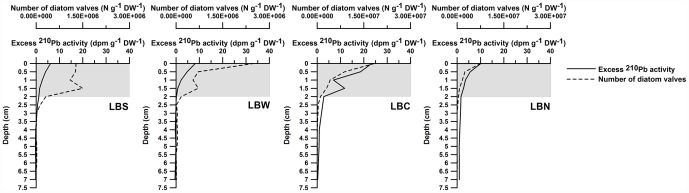
Number of diatom valves and excess ^210^Pb activity. Axis scale for diatom valves is different for Larsen B Central (LBC) and North (LBN).

Based on the ^14^C sediment accumulation rate (SAR) obtained for the Larsen sediment cores (∼0.04 cm y^−1^), only the upper millimeters of sediment were related to the post- ice-shelf collapse period [6], [7]. This low SAR, taken together with the correlation between the excess ^210^Pb activity and the abundance profiles of the diatom valves (**Fig. 4**) [6], indicates that diatoms reached the seabed after the collapse of the ice shelf and then were incorporated deeper into the upper 2 cm layer of the sediment column via diffusion (presumably bioturbation) rather than advection (**Fig. 2**). The presence of few valves below this layer is consistent with the theory that the biogenic silica reaching the seabed before the collapse of the ice shelf did not originate from diatoms.

In addition to diatoms, radiolarians, silicoflagellates and sponges are sources of biogenic silica in the marine environment [9]. In high-latitude sediments, diatom valves and sponge spicules can constitute major sources of this biogenic material on the seafloor [11], [9], [12], [13]. In the Antarctic continental shelf, sponges can show high abundances and biomasses [35], [23]. Eighty-one percent of Antarctic sponges belong to class Demospongiae, which are characterized by slow growth rates [36] and by a well-developed siliceous skeleton [37]. The absence of grounding ice under the Larsen B ice shelf most likely favored the development of Demospongiae in the EAP region [38], [26], leading to the relatively high percentage of this class in terms of biomass that was observed in previous studies of the Larsen regions [26], [39]. Sponges, similar to other suspension feeders, may develop under ice shelves [40]. Therefore, it is likely that sponge spicules from Hexactinellidae and Demospongiae accumulated in the Larsen B seafloor during the years of permanent shelf ice coverage. However, it has been observed that biogenic silica-rich sediments in regions with low productivity can be the result of the lateral transport of particles from adjacent areas [41], [10], [34]. Siliceous particles, including sponge spicule fragments, could have reached the Larsen region before the collapse of the ice shelf via lateral transport driven by the Weddell Gyre, which carries mud-like particles from the sponge-rich southeastern Weddell Sea [42] to the tip of the Antarctic Peninsula [43]. Further evidence of lateral transport into the Larsen region is provided by analyses of fatty acids, which were found below the upper 2 cm of the sediment column and had signals correlated to relatively older, refractory material [7].

Our results demonstrate that under ice shelves, the supply of biogenic silica from sponges (spicules) to the sedimentary record may be greater than that from diatomaceous sources (**Fig. 3**). However, these conditions are changing in the continental shelf below the extinct Larsen ice shelf. The concentration of biogenic silica originating from diatoms is becoming larger than that originating from spicules in the upper cm of the sediment column (**Fig. 3**), and this change is related to ongoing global warming, which is especially dramatic in the Antarctic Peninsula. This feature of the sedimentary record leaves unambiguous evidence of the disintegration of the ice shelf and provides further evidence of the effects of ongoing global warming in the Antarctic.

## Conclusions

The disintegration of the Larsen ice shelves produced a drastic change in the upper water column: it increased primary production and consequently increased the diatomaceous material. The arrival of this material shifted the sedimentary regime of biogenic silica from one dominated by sponge spicules to one in which diatom debris plays a more important role. This change will eventually integrate the region into the area of the Southern Ocean, where open water conditions during the austral summer create high levels of biogenic silica containing significant proportions of diatomaceous material. These results provide further evidence of the profound changes experienced in the benthic realm of the Antarctic as a consequence of ongoing climate change.
